# Can the Blended Application of Controlled-Release and Common Urea Effectively Replace the Common Urea in a Wheat–Maize Rotation System? A Case Study Based on a Long–Term Experiment

**DOI:** 10.3390/plants12244085

**Published:** 2023-12-06

**Authors:** Ling Zhang, Wen-Tao Xue, Hao Sun, Yun-Cai Hu, Rong Wu, Ye Tian, Yi-Shan Chen, Liang Ma, Qian Chen, Ying Du, Yang Bai, Shan-Jiang Liu, Guo-Yuan Zou

**Affiliations:** 1 Institute of Plant Nutrition, Resources and Environment, Beijing Academy of Agriculture and Forestry Sciences, Beijing 100097, China; 18854806552@163.com (L.Z.); xwtbj2014@163.com (W.-T.X.); yoyo1106@126.com (H.S.); 17310215735@163.com (R.W.); 82tianye@sina.com (Y.T.); cys8320@163.com (Y.-S.C.); maliangyouyou@sina.com (L.M.); c.q.123@163.com (Q.C.); doings@msn.cn (Y.D.); jiangxuehanshuang@hotmail.com (Y.B.); 2 Department of Plant Sciences, Chair of Plant Nutrition, Technical University of Munich, 85354 Freising, Germany; yc.hu@tum.de

**Keywords:** blending urea, wheat–maize rotation system, grain yield, soil n and c stocks, environmental impacts, ecosystem economic benefit

## Abstract

The one-time application of blended urea (BU), combining controlled-release urea (CRU) and uncoated urea, has proven to be a promising nitrogen (N) management strategy. However, the long-term sustainability of blending urea remains largely unexplored. To assess whether a single application of blended urea could effectively replace split uncoated urea applications, a long-term field experiment was conducted in the North China Plain (NCP). The results indicated that, when compared to common urea (CU) at the optimal N rate (180 kg N ha^−1^), BU achieved comparable grain yields, N uptake and NUE (61% vs. 62). BU exhibited a 12% higher 0–20 cm soil organic nitrogen stock and a 9% higher soil organic carbon (C) stock. Additionally, BU reduced life–cycle reactive N (Nr) losses and the N footprint by 10%, and lowered greenhouse gas (GHG) emissions and the C footprint by 7%. From an economic analysis perspective, BU demonstrated comparable private profitability and a 3% greater ecosystem economic benefit. Therefore, BU under the optimal N rate has the potential to substitute split applications of common urea in the long–term and can be regarded as a sustainable N management strategy for wheat and maize production in the NCP.

## 1. Introduction

Chemical nitrogen (N) fertilizer plays an extremely critical role in ensuring food security, while the excessive application of N fertilizers poses a significant threat to both the ecological environment and human health [1,2,3,4]. Urea is the most common form of N fertilizer, which accounts for 73% of N fertilizers that are globally applied [5]. When N fertilizer is overused, around 50% of the nitrogen (N) is released into the environment in diverse pollutant forms, such as nitrate (NO_3_^−^), ammonia (NH_3_) and nitrous oxide (N_2_O) [6,7]. These phenomena are even more serious in the winter wheat–summer maize rotation system in the North China Plain (NCP), which is an important agricultural production system and comprises more than 80% of the nationwide wheat production and more than 35% of the nationwide maize production [8]. Consequently, exploring a sustainable N management strategy is imperative to maximizing crop production while minimizing environmental impacts in China [9].

Numerous studies have explored optimal N management practices, including in-season root-zone N management, to closely align crop N uptake with soil N supply by splitting N applications. These N management strategies significantly improved grain yields and N-use efficiency (NUE), while reducing environmental risks [10,11]. However, it cannot be ignored that split applications of N fertilizer are usually not acceptable due to acute labor shortages and operation difficulties [12]. Recently, controlled-release urea (CRU) has attracted widespread attention worldwide, which could release N into soil at rates that closely match crops’ N demand, and thus could reduce the reactive nitrogen (Nr) losses and maximize NUE [13,14]. Additionally, a single application of CRU can save time and labor compared to split urea applications [15]. However, a prolonged N release rate from the CRU could result in inadequate N supply during the initial phases of crop growth [16], and the cost of CRU is higher than that of common uncoated urea [17].

A more effective N management strategy, blending urea (BU), which combines a controlled-release urea with conventional urea, could solve the above shortcomings, not only meeting the crop’s N demand throughout the entire growth period, but also reducing fertilization costs [18]. Some studies indicated that the one-time application of blending urea attained a comparable grain yield and higher NUE compared to the split application of common urea in the NCP [19,20]. Effective N management should apply the right N fertilizer source at the right rate and right time, and in the right place (“4R” principles) [21]. However, in these studies, the agronomically feasible N application rates were at a relatively high level (>210 kg ha^−1^), higher than the reported optimal N rate in the NCP (170 kg ha^−1^ and 172 kg ha^−1^ for wheat and maize, respectively) [22,23]. In addition, most of these studies focused on the effects of blending urea on grain yield and NUE only for 2–3 years. The long-term effects of mixing CRU and urea at an optimal N rate on grain yield and NUE under a wheat–maize rotation system remains unclear.

Sustainable agricultural development not only ensures food security, but also highlights the importance of reducing environmental impacts, maintaining or improving soil productivity, and increasing human welfare [24]. Among numerous environmental impact indicators, N and carbon (C) footprints, measured as Nr losses or GHG emissions per million grams of grain produced, represent widely adopted integrated factors, addressing the dual objectives of ensuring food security and promoting environmental sustainability [25,26]. Life-cycle assessment (LCA) is a “user-side” approach, which is primarily used to account for Nr releases and GHG emissions across the whole life-cycle of crop production [27]. The application of N fertilizer also affects soil quality (such as soil organic nitrogen (SON) and soil organic carbon (SOC) storage) by affecting crop growth, plant biomass C input to the soil, and microbial community composition and activity [28,29]. Long-term field experiments could determine changes in SOC storage and SON storage, and predict future changes in soil productivity to some extent [30]. The private profitability of farmers is the most direct factor representing the affordability and applicability of a specific N management approach [31]. In recent years, more and more studies have focused on the ecosystem economic benefit (EEB) [32,33], which comprehensively considers the benefits of crop yields and the costs regarding the environmental impact and human health derived from N fertilizer application [34]. Therefore, it is imperative to consider both the private profitability of farmers and the economic benefits to the ecosystem to promote sustainable crop production. However, the long-term and integrated effects of the one-time application of blending urea on environmental impacts, private profitability and ecosystem economic benefit, as well as soil quality, remain unclear.

The objectives of this study were to explore whether the one-off application of blending urea can effectively replace the split application of common urea by analyzing the comprehensive performance of agronomic impacts (grain yield, N uptake and NUE), environmental impacts (Nr losses, GHG emissions, N and C footprints), soil fertility (SON storage and SOC storage), and economic benefits (private profitability and ecosystem economic benefit). We hypothesize that the one-time application of blending urea at the optimal N rate could replace common urea for a long time by maintaining or even improving agronomic, soil fertility, environment impacts, and economic performance. The results will provide scientific support for the development and improvement of environmentally friendly and agronomically viable N fertilizer products for crop production.

## 2. Results

### 2.1. Grain Yield, N Uptake and N Use Efficiency

**Figure 1:**
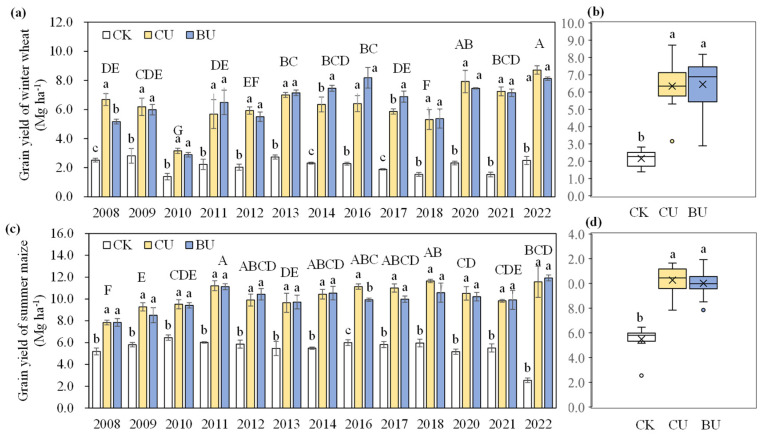
Annual winter wheat yield (**a**), mean winter wheat yield (**b**), annual summer maize yield (**c**), and mean maize yield (**d**) under different N treatments during 2008–2022. CK, no fertilizers; CU, common urea; BU, blending CRU with common urea. Vertical bars represent ±S.E. of the mean in (**a**,**c**). The horizontal black line and multiple signs in the box of (**b**,**d**) indicate median and mean values, respectively, and the bars of the box indicate the upper and lower quartiles. Small letters that are the same do not exhibit significant differences among various N treatments at *p* < 0.05; capital letters that are the same do not exhibit significant differences among years at *p* < 0.05, as determined by LSD.

From 2008 to 2022, N fertilizer application significantly increased the grain yield (Figure 1, Table S1). In the winter wheat season, the grain yield increased from an average of 2.2 Mg ha^−1^ under CK treatment to an average of 6. 3 Mg ha^−1^ under CU and 6.4 Mg ha^−1^ under BU treatment (Figure 1b). Similarly, for summer production, the grain yields of CU (10.3 Mg ha^−1^) and BU (10.0 Mg ha^−1^) were also significantly higher than that of CK treatment (5.5 Mg ha^−1^) (Figure 1d). In most years, there was no significant difference in grain yield between CU and BU treatment, except that the grain yield of winter in BU was 17.5% and 17.6% greater than that of CU in 2014 and 2017, respectively; BU treatment achieved a 22.7% lower grain yield of wheat in 2008 and 10.6% lower grain yield of maize in 2016 compared to CU treatment (Figure 1a,c).

During 2008–2022, the average N uptake for winter wheat, summer maize and the whole rotation system showed the same trend response to N management strategies (Table 1). The results reflected that BU and CU treatments achieved significantly higher N uptake than CK, and no significant difference in mean nitrogen (N) uptake was observed between CU and BU treatments (Table 1, Table S2). Similarly, there is no significant difference in NUE in the rotation system between CU (62%) and BU (61%) treatments.

### 2.2. Soil N and C Stocks Dynamics

**Figure 2:**
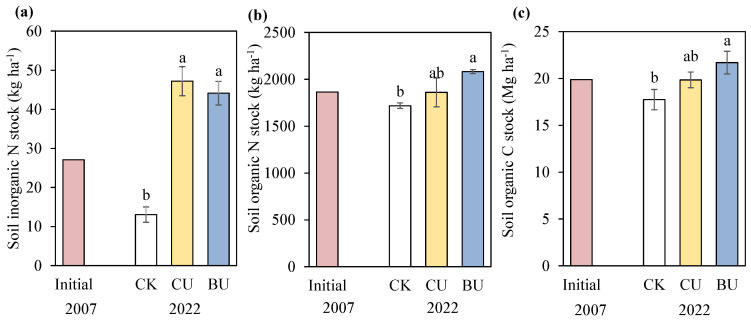
Soil inorganic N stock (**a**), soil organic N stock (**b**), and soil organic C stock (**c**) at 0–20 cm soil depth under different N treatments in 2022. CK, no fertilizers; CU, common urea; BU, BU, blending CRU with common urea. Vertical bars represent ±S.E. of the mean. Small letters that are the same do not exhibit significant differences among various N treatments at *p* < 0.05, as determined by LSD.

In 2022, the 0–20 cm soil inorganic N stock did not significantly differ between CU (47 kg ha^−1^) and BU (44 kg ha^−1^) treatments, but significantly increased compared to that in the CK treatment (13 kg ha^−1^) (Figure 2a, Table S3). N fertilizer application significantly increased the 0–20 cm SON stock and SOC stock, which showed similar trends: BU > CU > CK. When compared to CU, BU obtained a 12% greater SON stock (2081 vs. 1860 kg ha^−1^) and 9% greater SOC stock (21.7 vs. 19.8 Mg ha^−1^) (Figure 2b,c).

Compared to the initial values in 2007, long-term conditions without N fertilizer application consumed 52% of the soil inorganic N stock, 8% of the SON stock and 11% of the SOC stock (Figure 2b,c). After the long-term application of common urea under CU treatment, the soil inorganic N stock increased by 74%, while the SON stock and SOC stock remained basically unchanged. The long-term application of blending urea enhanced 63% of the soil inorganic N stock (44 vs. 27 kg ha^−1^), 12% of the SON stock (2081 vs. 1862 kg ha^−1^), and 9% of the SOC stock (21.7 vs. 19.9 Mg ha^−1^).

### 2.3. Nr Losses, GHG Emissions, N and C Footprint

**Figure 3:**
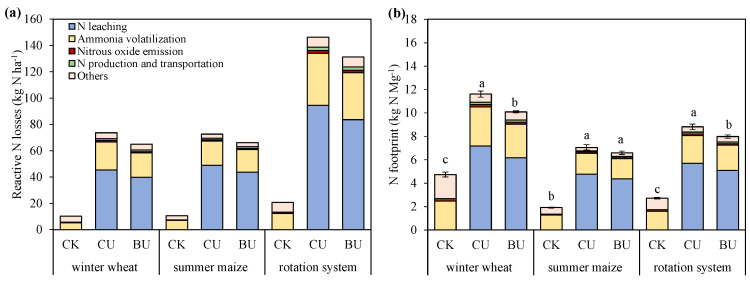
Average reactive N losses (**a**) and average N footprint (**b**) at the life-cycle scale under different N treatments during 2008–2022. CK, no fertilizers; CU, common urea; BU, BU, blending CRU with common urea. Vertical bars represent ±S.E. of the mean. Small letters that are the same do not exhibit significant differences among various N treatments at *p* < 0.05, as determined by LSD.

In the winter wheat season, summer maize season and the whole rotation system, both the average life-cycle Nr losses and N footprint increased in BU and CU treatments compared to CK treatment (Figure 3). BU demonstrated an 11%, 9%, and 10% reduction in life-cycle Nr losses compared to CU for the winter wheat season, summer maize season and the whole rotation system. Also, BU significantly reduced the N footprint by 13% (9.2 vs. 10.7 kg N Mg^−1^) and 10% (7.4 vs. 8.2 kg N Mg^−1^) for the winter wheat season and the whole rotation system, while no significant difference was observed in N footprint between BU and CU treatment for the maize season (6.2 vs. 6.7 kg N Mg^−1^) (Table S4). In fertilized treatments, the largest contributor to Nr losses and N footprint was N leaching, with the contribution proportion of 61–68%, followed by NH_3_ volatilization (25–29%).

**Figure 4:**
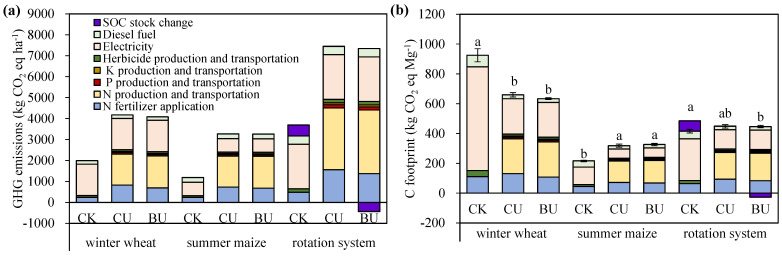
Average GHG emissions (**a**) and C footprint (**b**) at the life-cycle scale under different N treatments during 2008–2022. CK, no fertilizers; CU, common urea; BU, BU, blending CRU with common urea. Vertical bars represent ±S.E. of the mean. Small letters that are the same do not exhibit significant differences among the various N treatments at *p* < 0.05, as determined by LSD.

Compared to CK treatment, the life-cycle GHG emissions increased in BU and CU treatments; C footprint showed a decreased trend under BU and CU treatments in wheat seasons and the whole rotation system, while it increased under fertilized treatments in maize seasons (Figure 4, Table S5). The different trends in the C footprint originated from the difference in yields between wheat and maize under CK treatment. When no N fertilizer was applied, as a C4 crop, maize can achieve a higher yield than wheat, resulting in a lower C footprint in maize. Compared to CU, the use of BU resulted in a 3% increase in GHG emissions associated with the production and transportation of N fertilizer. However, BU decreased GHG emissions resulting from N fertilizer application by 16%, 7%, and 12% during the winter wheat season, the summer maize season, and the entire rotation system, respectively. From the perspective of the entire life-cycle process, BU exhibited comparable GHG emissions and a comparable C footprint in winter and maize seasons to CU, while it had 7% lower GHG emissions (6903 vs. 7457 kg CO_2_ eq ha^−1^) and 7% smaller C footprint (420 vs. 449 kg CO_2_ eq Mg^−1^) throughout the whole rotation system (Figure 4).

In the applied N fertilizer treatments, 54–68% of the GHG emissions and C footprint were associated with the N fertilizer, including the manufacture, transportation and on-farm application process (Figure 4). It is noteworthy that the electricity used for irrigation was also an important contributor (with a share of 20–37%), especially in the high-water-consumption wheat season. When evaluating the GHG emissions and C footprint of the entire rotation system, soil carbon changes are taken into account. In CK treatment, the reduced soil C stock (converted to 524 kg CO_2_ eq ha^−1^) contributed 14% of GHG emissions and C footprint. In BU treatment, 440 kg CO_2_ eq ha^−1^ were sequestered in the soil, mitigating 6% of GHG emissions and C footprint.

### 2.4. Economic Benefit Analysis

In the whole rotation system, the average N-derived grain benefits during 2008–2022 were comparable in CU and BU treatments (Table 2). Compared to CU, one-off BU application raised the N fertilizer cost by 9% (244 vs. 223 USD ha^−1^), while saving 50% of labor costs (85 vs. 171 USD ha^−1^) during N fertilizer application. Ultimately, BU obtained comparable private profitability (2836 vs. 2812 USD ha^−1^) (Table 2). The combined total of ecological and health costs constituted 14–16% of the corresponding grain yield benefits, surpassing the percentage represented by N fertilizer and labor costs (10–13%). Thus, the costs from ecological damage and human health could not be ignored in a sustainable N management strategy [34]. Compared to CU treatment, one-off BU application reduced ecological costs and health costs by 10%. In aggregating the costs associated with N fertilizer, labor, ecosystem, and human health, one-off BU application improved the EEB by 3% compared to CU (2386 vs. 2313 USD ha^−1^) (Table 2).

## 3. Discussion

### 3.1. Grain Yield, N Uptake and N-Use Efficiency

In the current investigation, we assessed the agronomic performance of two N management strategies. Our findings indicate that, at the optimal N rate, blending urea maintained a similar grain yield, N uptake and NUE when compared to CU across most years (Figure 1, Table 1). These results suggested the viability of a one-time application of CRU mixed with an uncoated urea, serving as an alternative to the split application of urea, ensuring both crop production and efficient resource utilization. Previous studies have shown that CRU mixed with urea achieved approximately the same yield as common urea, but significantly increased NUE by 4.7–26.6% [35]. The different results regarding NUE may be because NUE in the previous study was at a relatively low level compared to this article (34.7–44.0% vs. 61–62%). The yield level of this study is relatively high, and higher than the national average yield (5.4 Mg ha^−1^ for wheat and 6.1 Mg ha^−1^ for maize) [8]. Due to differences in climate conditions from year to year, grain yield fluctuates, emphasizing the importance of long-term field experiments capable of taking into account the changing climate conditions [21]. Furthermore, the NUE level throughout the entire crop rotation system is close to the national target and the NUE level in the United States (68%), where the N fertilizer rate is lower than the crop N uptake [36,37].

### 3.2. Soil N and C Stocks Dynamics

In addition to crop absorption and Nr loss, a portion of N from fertilizer and environmental sources is either retained in the soil as residual inorganic N or converted into SON pools [38]. The continuous augmentation of SON pools contributes to a stable inherent nitrogen supply in the soil, diminishing the need for N fertilizers and enhancing NUE [39]. SOC serves as another pivotal indicator of soil fertility and stands as a cornerstone in sustainable agricultural development, which significantly influences soil physical properties and microbial activity [40,41]. This study demonstrated that the prolonged application of mixed urea enhanced surface soil fertility, exhibiting a 12% higher SON stock and a 9% higher SOC stock compared to common urea (Figure 2). Previous research has shown that the long-term application of blended urea significantly improved surface soil fertility, although no noteworthy difference was discerned in subsurface layers [19]. Similarly, the controlled-release of N fertilizer was considered an effective measure to improve SOC storage, significantly increasing SOC storage in small macro-aggregates (0.25–2 mm) [42]. This disparity in impact may be attributed to the CRU’s ability to regulate the release rate of N fertilizer, thereby extending the release period of N fertilizer with a low soil N concentration. This, in turn, enhanced soil C and N’s energy resources and microbial metabolic activity, ultimately promoting the accumulation of soil organic nitrogen and carbon [43,44]. However, further research is imperative to comprehend the microbial mechanisms underlying the long-term application of mixed urea in enhancing both SON and SOC stocks.

### 3.3. Nr Losses, GHG Emissions, N and C Footprint

This study demonstrated that BU showed significant advantages in mitigating Nr losses and reducing the N footprint, primarily attributed to the diminished N leaching (10–12%) and NH_3_ volatilization (6–12%) (Figure 3). Similar findings were reported by Xia et al. [45], who found that the combination of CRU with optimized N application rates significantly reduced N losses through various pathways. CRU was designed to release N steadily, promoting synchronization between soil N supply and crop N demand, consequently reducing substrate concentration and shortening exposure time, and thereby minimizing NH_3_ volatilization and N leaching [46,47,48]. Although this study incorporated the most widely used Nr emission factors and site-specific Nr loss empirical models to ensure the accuracy of results, some uncertainties were unavoidable. Future research should consider measuring N loss under field conditions for a more comprehensive assessment.

In the process of nitrogen fertilizer production and transportation, BU treatment increased GHG emissions by 3% compared to CU treatment. This increase was due to the fact that producing 1 kg of CRU emitted 0.72 kg more CO_2_ than the production of 1 kg of conventional urea [49]. However, during the field application of N fertilizer, BU substantially reduced GHG emissions by an amount large enough to compensate for the increase in GHG emissions from CRU fertilizer production (Figure 4). Ultimately, across the entire life-cycle scale, BU showed comparable or lower GHG emissions and a comparable or smaller C footprint. Research has shown that the C footprint for wheat and maize production in China is 900 and 600 kg CO_2_ Eq. Mg^−1^ [50], which is higher than the C footprint in N-applied treatments in this study. The main reason for this difference is that the yield level in this study was higher than the average yield level in China. The utilization of N fertilizers has been extensively documented as a significant contributor to GHG emissions in the LCA of agricultural production, emphasizing the importance of optimizing N application rates to reduce GHG emissions [51].

Soil carbon has a dual impact on greenhouse gas emissions [52]. On one hand, the reduced SOC pool is the source of CO_2_ emissions; for example, in CK treatment, the soil C stock was reduced, which contributed 14% of GHG emissions and the C footprint. On the other hand, soil serves as a carbon sink, fixing a portion of CO_2_ emissions in the soil. In BU treatment, the increasing SOC pool mitigated 6% of GHG emissions and C footprint. Therefore, improving SOC in agriculture systems is important for offsetting anthropogenic GHG emissions [53]. Overall, the blended application of controlled-release and uncoated urea to reduce the N footprint and C footprint for intensive wheat and maize production in NCP is promising, stemming from the significant SOC accumulation, and decreased various Nr loss.

### 3.4. Economic Benefit Analysis

The primary and most effective motivation for farmers to adopt N management strategies is pursuing increased private profitability. Moreover, the EEB serves as an effective indicator of the ability to reconcile the trade-offs between crops’ productivity and environmental risks, assessing whether EEB is essential to the development of new N management strategies [23]. In the current study, one-off BU application in conjunction with an optimal N rate not only improved EEB but also preserved private profitability for farmers at levels comparable to CU (Table 2); this could be viewed as a mutually beneficial N management strategy for both farmers and public health. Due to the ongoing urbanization in China, an increasing number of farmers are migrating from rural regions, which has led to a labor shortage and escalated labor expenses [51]. Blending urea aligns with this prevailing trend in China’s development and promises enhanced economic advantages in the foreseeable future.

### 3.5. Sustainable N Management Strategy: Blending Urea Applied at Optimal N Rate

**Figure 5:**
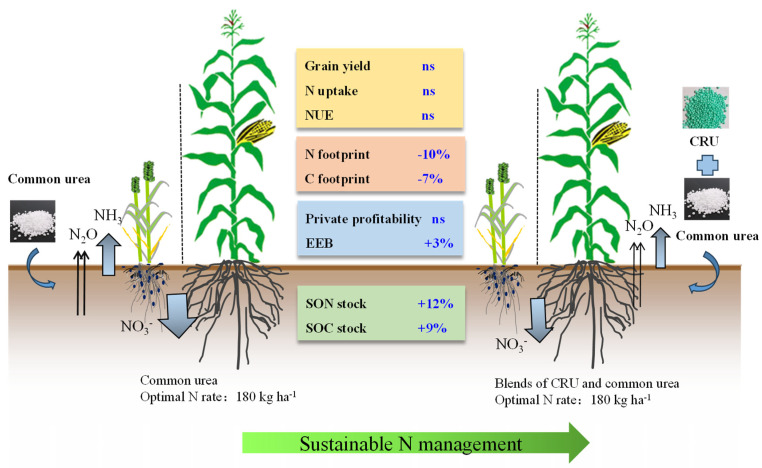
Sustainable N management strategy: blending urea applied at optimal N rate. The abbreviation “ns” signifies the absence of a statistically significant difference between the two nitrogen (N) management strategies.

Sustainable N management strategies must follow the 4R guidelines: the right rate, right source, right time and right place [21]. The application of blended urea at the optimal N rate aligns with the 4R guidelines, with a particular focus on adhering to the “right source” and “right rate” aspects. Compared to common urea, blended urea is considered an improved “Right source”, which could closely match soil N supply and crop N demand throughout the entire growth period by controlling the rate of N release [23]. For the “Right rate”, 180 kg N ha^−1^ is close to the optimal N rate for wheat and maize in the NCP, as reported by previous studies [22,23]. In the present study, blending urea applied at an optimal N rate demonstrated the capacity to effectively balance the trade-offs between crop production, soil fertility, economic benefits, and environmental impacts (Figure 5). For wheat and maize production in the NCP, the one-time application of BU could replace the split applications of common urea in the long-term. The findings of this study also provided scientific guidelines for the design of site-specific, sustainable N management strategies. More case studies should be carried out to facilitate the widespread adoption of blending urea at the optimal N rate across different regions and crop systems. This should be achieved by meticulous consideration of local climate conditions, soil properties, crop N demand, and field management practices.

## 4. Materials and Methods

### 4.1. Experiment Site, Experimental Design, and Field Management

**Figure 6:**
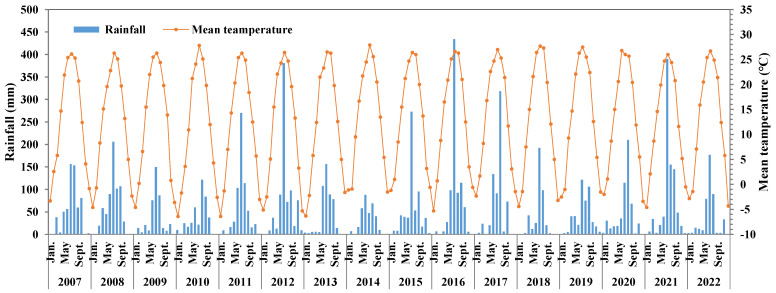
Changes in mean air temperature and rainfall during 2008–2022.

A long-term (from 2007 to 2022) field experiment with a winter wheat–summer maize rotation was conducted in Fangshan District (36°40′ N, 116°08′ E, elevation 39.2 m), Beijing Municipality, Northern China. The study area has a continental monsoon climate. The annual average temperature is 12.6 °C and the annual rainfall is 608 mm, with about 80% of precipitation occurring during June–August (Figure 6). The field experiment began with the winter wheat season. Winter wheat is sown in mid-October and is harvested in early June; then, the summer maize is sown. The soil type is fluvo-aquic soil and the soil texture is clay loam. The basic physical–chemical properties of the initial top soil layer (0–20 cm) were as follows: pH 8.21, bulk density 1.27 g cm^−3^, organic matter content 12.6 g kg^−1^, alkali-hydrolyzale nitrogen (N) 58.1 mg kg^−1^, available phosphorus (Olsen-P) 6.04 mg kg^−1^, available potassium (K) 56.4 mg kg^−1^, total N 0.75 kg^−1^, total P 0.82 g kg^−1^, and total K 17.4 g kg^−1^.

The field experiment had a randomized block design with three N treatments and three replicate plots (10 m × 8 m) per treatment. The three N treatments included: no N fertilizer application (CK); uncoated urea (46% N, CU), as used by split fertilization; blends of CRU and common urea (blending urea, BU) in 1:2 ratio (60 kg ha^−1^ N rate from CRU and 120 kg ha^−1^ N rate from common urea), which was applied as basal fertilizer. The CRU in this study is the resin-coated urea (45% N), which has a two-month N-release longevity at 25 °C. The N rate for CU and BU was 180 kg ha^−1^, which was considered the optimal N rate for wheat and maize in the NCP [22,23]. For CU treatment, 120 kg N ha^−1^ uncoated urea was applied initially, before planting, and 60 kg N ha^−1^ uncoated urea was top-dressed at the stem elongation stage in the wheat season and at the 10-leaf stage in the maize season.

Phosphate and potassium fertilizers in CU and BU were applied once as basal fertilizers at 90 kg ha^−1^ (P_2_O_5_) and 90 kg ha^−1^ (K_2_O) in both wheat and maize seasons, which were broadcasted and concurrently incorporated into the upper soil layer along with the N fertilizer. After the wheat and maize were harvested, the remaining residues of wheat and maize were removed from the field. The irrigation volume varied with annual rainfall; about 80 mm of irrigation water was applied 2–4 times in the wheat season and 1–2 times in the maize season. Pesticides were applied to control disease, weeds and insects.

### 4.2. Measurements and Evaluation Indicators

#### 4.2.1. Sampling and Chemical Analysis

At the harvest stage, the wheat and maize in a 2-m^2^ area were manually harvested, oven-dried and weighed to determine standard grain yield (14% water content for wheat and 15.5% water content for maize). The N uptake was determined by multiplying nitrogen concentration by biomass, as in a previous study [22]. The apparent recovery N-use efficiency (NUE) was defined as the N uptake difference between fertilized and unfertilized control plots divided by N rate [38].

After the maize harvest in 2022, soil samples were collected at depths of 0–20 cm, then air-dried, and passed through a 0.25 mm sieve for further analysis. The soil concentration of inorganic N was analyzed using an autoanalyzer (Model AA3-A001-02E, Bran-Luebbe, Norderstedt, Germany). Soil samples were acid-washed and analyzed for SOC and soil total N concentration contents by dry combustion using an elemental analyzer (Vario Macro CNS analyzer, Elementar, Langenselbold, Germany). The soil organic N (SON) content was equal to the total nitrogen content minus the inorganic nitrogen content. The soil inorganic N stock, SON stock and SOC stock were calculated according to Novara et al. [53] as follows:

Soil inorganic N stock (kg ha^−1^) = soil inorganic N concentration (mg kg^−1^) × bulk density (Mg m^−3^) × depth (m) × 10 (1)

SON stock (kg ha^−1^) = SON concentration (g kg^−1^) × bulk density (Mg m^−3^) × depth (m) × 10^4^ (2)

SOC stock (Mg ha^−1^) = SOC concentration (g kg^−1^) × bulk density (Mg m^−3^) × depth (m) × 10 (3)

There was no significant difference in bulk density among different N treatments, with an average of 1.26 Mg m^−3^.

#### 4.2.2. Environmental Impacts Calculations

The system boundary was delineated to encompass the production of winter wheat and summer maize, from the production and transportation of agricultural materials to the farm gates, arable farming operations and crops’ harvest. The Nr losses and the GHG emissions were evaluated using the LCA method [54,55]. The Nr emissions (including N leaching, NH_3_ volatilization and nitrous oxide (N_2_O) emissions) could be divided into three sources: (1) the application of N fertilizer; (2) the manufacture and transportation of N fertilizer; (3) other agricultural materials’ inputs, including the manufacture and transportation of P and K fertilizer, pesticides, diesel fuel, and electricity for irrigation. The Nr emissions were calculated according to Equation (4), as follows:

(4) $$\mathrm{Nr}\;\mathrm{emission}=\sum_{i=1}^{m}{\mathrm{Rate}}_{i}\times {\mathrm{EF}}_{i}\;+\;N_{2}O_{\mathrm{direct}}\;+\;N\;\mathrm{leaching}\;+\;{\mathrm{NH}}_{3}\;\mathrm{volatilization}$$

where *i* (=1, 2, 3…) denote different agricultural materials. Rate*_i_* and EF*_i_* represent the amount of agricultural materials used per hectare and the corresponding Nr emission factors of agricultural materials, respectively, which are listed in Tables S6 and S7. N_2_O_direct_ represents the direct N_2_O emissions.

To ensure the accuracy of Nr losses, the empirical models and emission factor of Nr losses under similar climate conditions were selected in this study. The Nr losses from common urea and CRU were calculated using Equations (5)–(8) according to Zhang et al. [23] and Liang [56]:

NH_3_-N_urea_ in wheat = 8.8% × N_urea_ rate + 5.3 (5)

NH_3_-N_CRU_ in wheat = 4.3% × N_CRU_ rate + 5.3 (6)

N_2_O_direct_-N_urea_ in wheat = 0.46 × e^(0.0054 × N-urea rate)^ (7)

N_2_O_direct_-N_CRU_ in wheat = 0.46 × e^(0.0037 × N-CRU rate)^ (8)

NH_3_-N_urea_ in maize = 6.5% × N_urea_ rate + 7.0 (9)

NH_3_-N_CRU_ in maize = 4.5% × N_CRU_ rate + 7.0 (10)

N_2_O_direct_-N_urea_ in maize = 0.31% × N_urea_ rate + 0.45 (11)

N_2_O_direct_-N_CRU_ in maize = 0.21% × N_CRU_ rate + 0.45 (12)

The N leaching factor for urea and CRU were calculated to be 26.9% and 16.9% for wheat, and 27.2% and 18.4% for maize [52]. The N_2_O_direct_, N leaching and NH_3_ volatilization of BU treatment were the sum of the common urea and CRU.

During the whole life-cycle, the total amount of GHG emissions expressed in carbon dioxide equivalents per hectare included CO_2_ and N_2_O emissions from (1) arable lands after N fertilizer use, (2) the manufacture and transportation of N fertilizer, (3) the production and transportation of other agricultural inputs, (4) changes in soil organic carbon stock. Thus, GHG emissions were calculated as follows:

(13) $$\mathrm{GHG}\;\mathrm{emissions}=\sum_{i=1}^{m}{\mathrm{Rate}}_{i}\times {\mathrm{EF}}_{i}\;+\;N_{2}O_{\mathrm{total}}\;-\;N\;\times \;44/28\;\times \;298\;+\;\mathrm{annual}\mathrm{reduction}\mathrm{in}\mathrm{SOC}\mathrm{stock}\;\times \;44/12$$

N_2_O_total_–N = N_2_O_direct_ − N + 1% × NH_3_ − N + 0.75% × NO_3_^−^ − N (14)

where *i* (=1, 2, 3…) denotes various agricultural materials. Rate*_i_* and EF*_i_* represent amount of applied agricultural materials and the GHG emission factors from the manufacture and transportation of agricultural materials, respectively. EF*_i_* and Rate*_i_* are listed in Tables S1 and S2. N_2_O_total_–N was Nr emitted as N_2_O from the soils, including the direct pathway and indirect pathways [57]. The N footprint (kg N Mg^−1^) and C footprint (kg CO_2_ eq Mg^−1^) were defined as the Nr emission and GHG emissions during the entire life-cycle per million grams of harvested grain production.

#### 4.2.3. Economic Benefits Analysis

Given the fact that the N management strategy was the only source of the difference in economic benefits between BU and CU, this study exclusively concentrated on the costs and economic benefits associated with N fertilizer. Private profitability and ecosystem economic benefit (EEB) were estimated to evaluate the monetary value for producers and public interests, respectively. The private profitability and EEB (USD ha^−1^) were calculated using Equations (15)–(16) according to Zhang et al. [23]:

Private profitability = N-derived grain × W_price_ − N_cost_ − L_cost_ (15)

EEB = N-derived grain × W_price_ − N_cost_ − L_cost_ − E_cost_ − H_cost_ (16)

where N-derived grain was the grain difference between N fertilized and CK treatment; W_price_ were the market price of wheat and maize, which averaged 0.4 and 0.32 USD kg^−1^. The N_cost_ and L_cost_ denote the costs of the N fertilizer and labor for N fertilizer application. The N_cost_ was determined by multiplying the N rate by N fertilizer prices (0.62 USD kg^−1^ for uncoated urea and 0.79 USD kg^−1^ for CRU). A total of 43 USD ha^−1^ for one-time N fertilizer application was used here for labor prices. E_cost_ and H_cost_ represent ecosystem damage costs and human health costs, respectively, caused by various Nr releases or GHG emissions in N fertilizer production and application. The detailed calculation of E_cost_ and H_cost_ was carried out according to Ying et al. [34] and Zhang et al. [23].

### 4.3. Statistical Analysis

A two-way ANOVAs model with N management strategies (df = 2) and years (df = 12) was used to assess the overall variability of grain yield. A one-way analysis of variance (ANOVA) model with N management strategies (df = 2) as the factor was used to evaluate the overall variability in N uptake, NUE, soil N and C stocks and environmental impacts. When an ANOVA was significant, the least significant difference (LSD) test at the 0.05 probability level was used to compare the means of treatment. SAS software (ver. 6.12; SAS Institute, Cary, NC, USA) was used to conduct the analyses.

## 5. Conclusions

The one-time blended application of CRU and uncoated urea at the optimal N rate showed multifaceted advantages compared to common urea: (1) it maintained a relatively high crop yield and NUE, (2) improved the SON stock by 12% and SOC stock by 9%, (3) markedly mitigated life-cycle environmental impacts, notably reducing the N footprint by 10% and C footprint by 7%, and (4) improved the ecosystem economic benefits by 3%. In conclusion, BU under the optimal N rate can be chosen as a sustainable N management strategy, and can replace the split applications of common urea to boost sustainable wheat and maize production in the NCP in the long-term. In the future, more case studies should be carried out to explore an optimal formula of BU that is suitable for different ecological regions and crop systems.

## Figures and Tables

**Table 1:** Average aboveground N uptake of winter wheat, summer maize and the whole rotation system, and N-use efficiency (NUE) under various N treatments during 2008–2022.

Treatment	Average N Uptake (kg ha^−1^)			NUE in Rotation System (%)
	Winter Wheat	Summer Maize	Rotation System	
CK	62 b	86 b	148 b	/
CU	182 a	191 a	373 a	62% a
BU	185 a	184 a	369 a	61% a

CK, no fertilizers; CU, common urea; BU, blending CRU with common urea. Small letters that are the same do not exhibit significant differences among various N treatments at *p* < 0.05, as determined by LSD.

**Table 2:** The economic evaluation of winter wheat–summer maize rotation system under varying N treatments.

Treatment	N-Derived Grain Benefits	N Costs	Labor Costs	Ecological Costs	Health Costs	Private Profitability	Ecosystem Economic Benefit
		(USD ha^−1^)					
CU	3206	223	171	349	150	2812	2313
BU	3165	244	85	315	135	2836	2386

CK, no fertilizers; CU, common urea; BU, blends of CRU and common urea.

## Data Availability

The data presented in this study are available on request from the corresponding author.

## Supplementary Materials

The following supporting information can be downloaded at: https://www.mdpi.com/article/10.3390/plants12244085/s1, Table S1: ANOVA table on the effects of N management strategies and years on grain yield; Table S2: ANOVA table on the effects of N management strategies on N uptake; Table S3: ANOVA table on the effects of N management strategies on soil C inorganic N stock, soil organic N stock and soil organic C stock; Table S4: ANOVA table on the effects of N management strategies on N footprint; Table S5: ANOVA table on the effects of N management strategies on C footprint; Table S6: Reactive N emissions and GHG emissions factors for production and transportation of various agricultural inputs in the winter wheat-summer maize system; Table S7: Mean application rate of various agricultural inputs in 2008−2022 in the winter wheat-summer maize system. References [58,59,60,61,62,63] are cited in the Supplementary Materials.
