# Supplementary material for: Can the Blended Application of Controlled-Release and Common Urea Effectively Replace the Common Urea in a Wheat–Maize Rotation System? A Case Study Based on a Long–Term Experiment
Source: Plants (Basel). 2023 Dec 6;12(24):4085. doi: 10.3390/plants12244085 (PMC10748289; doi:10.3390/plants12244085)
Supplement: Supplementary file 1 [file plants-12-04085-s001.zip › plants-2719621-supplementary.pdf]

Supplementary information

Can the Blended Application of Controlled-Release and Common Urea Effectively Replace the Common Urea in a Wheat-Maize Rotation System? A Case Study Based on a Long-Term Experiment

Ling Zhang[1,†], Wen-Tao Xue[1,†], Hao Sun[1], Yun-Cai Hu[2], Rong Wu[1], Ye Tian[1], Yi-Shan Chen[1], Liang Ma[1], Qian Chen[1], Ying Du[1], Yang Bai[1], Shan-Jiang Liu[1,*], Guo-Yuan Zou[1,*]

1. Institute of Plant Nutrition, Resources and Environment, Beijing Academy of Agriculture and Forestry Sciences, Beijing 100097, China; 18854806552@163.com (L.Z.); xwtbj2014@163.com (W.-T.X.); yoyo1106@126.com (H.S.); 17310215735@163.com (R.W.); 82tianye@sina.com (Y.T.); cys8320@163.com (Y.-S.Z.); maliangyouyou@sina.com (L.M.); c.q.123@163.com (Q.C.); doings@msn.cn (Y.D.); jiangxuehanshuang@hotmail.com (Y.B.); liushanjiang@263.net (S.-J.Z.); gyzou@163.com (G.-Y.Z.)
2. Department of Plant Sciences, Chair of Plant Nutrition, Technical University of Munich, 85354 Freising, Germany; yc.hu@tum.de (Y.-C.H)

† These authors contributed equally to this work.

*Correspondence: liushanjiang@263.net; gyzou@163.com

Supplemental Tables. S1-S7

Table S1

ANOVA table on the effects of N management strategies and years on grain yield.

	Source	df	Anova SS	Mean Square	F value	P value
Wheat	Year	12	46.1	3.8	6.25	<0.0001
	N	2	565.9	282.9	461.39	<0.0001
	Year*N	24	68.1	2.8	4.63	<0.0001
Maize	Year	12	103.1	8.6	17.89	<0.0001
	N	2	466.4	233.2	485.47	<0.0001
	Year*N	24	45.7	1.9	3.96	<0.0001

Table S2

ANOVA table on the effects of N management strategies on N uptake.

	Source	df	Anova SS	Mean Square	F value	P value
N uptake in wheat	N	2	29588	14794	1028.47	<0.0001
N uptake in maize	N	2	29588	14794	1028.47	<0.0001
N uptake in rotation system	N	2	99738	49869	335.57	<0.0001

Table S3

ANOVA table on the effects of N management strategies on soil C inorganic N stock, soil organic N stock and soil organic C stock.

	Source	df	Anova SS	Mean Square	F value	P value
soil inorganic N stock	N	2	2142.4	1071.2	40.72	0.0003
soil organic N stock	N	2	20157.1	100782.6	4.14	0.0741
soil organic C stock	N	2	23.4	11.7	3.60	0.0939

Table S4

ANOVA table on the effects of N management strategies on N footprint.

	Source	df	Anova SS	Mean Square	F value	P value
N footprint in maize	N	2	78.2	39,1	341.66	<0.0001
N footprint in wheat	N	2	48.4	24.2	315.35	<0.0001
N footprint in rotation system	N	2	65.6	32.8	436.8	<0.0001

Table S5

ANOVA table on the effects of N management strategies on C footprint.

	Source	df	Anova SS	Mean Square	F value	P value
C footprint in maize	N	2	156382	78191	40.55	0.0003
C footprint in wheat	N	2	22685	11342	61.51	0.0001
C footprint in rotation system	N	2	6344	3172	8.97	0.0157

Table S6

Reactive N emissions and GHG emissions factors for production and transportation of various agricultural inputs in the winter wheat-summer maize system.

Item	Unit	Reactive N emission (10^{-3} kg N unit^{-1})	GHG emission (kg CO_2 eq unit^{-1})
Production and transportation of urea	kg N	7.15	8.30
Production and transportation of CRU*	kg N	7.15	9.02
Production and transportation of P fertilizer	kg P_2O_5	0.184	0.79
Production and transportation of K fertilizer	kg K_2O	0.146	0.55
Production and transportation of herbicide	kg	4.69	19.13
Electricity used for irrigation	kWh	1.97	0.95
Diesel fuel	L	2.86	3.75

*Compared with urea, the production and transportation of CRU mainly increased the electricity and diesel fuel consumption, resulting in 0.72 kg CO_2 eq more GHG emission for 1 kg CRU; while the reactive N losses difference between two N fertilizer could be negligible.

Table S7

Mean application rate of various agricultural inputs in 2008-2022 in the winter wheat-summer maize system.

	P fertilizer (kg P_2O_5 ha^{-1})	K fertilizer (kg K_2O ha^{-1})	Pesticides (kg ha^{-1})	Diesel fuel (L ha^{-1})	Electricity for irrigation (kWh)
winter wheat	90	90	4.5	45	1575*
summer maize	90	90	3.8	60	675*

*value was the electricity used for irrigation, which was calculated by the irrigation electricity use quantity (450 kWh per irrigation in the NCP) multiplied by average irrigation time (3.5 time in wheat season and 1.5 times in maize season).

Reference

Clark, S.; Khoshnevisan, B.; Sefeedpari, P. Energy efficiency and greenhouse gas emissions during transition to organic and reduced-input practices: Student farm case study. *Ecol. Eng.* **2016**, 88, 186–194.

Cui, Z.; Yue, S.; Wang, G.; Meng, Q.; Wu, L.; Yang, Z.; Zhang, Q.; Li, S.; Zhang, F.; Chen, X. In-season root-zone N management for mitigating greenhouse gas emission and reactive N losses in intensive wheat production. *Environ. Sci. Technol.* **2013**, 47, 6015–6022.

Huang, J.; Chen, Y.; Sui, P.; Gao, W. Estimation of net greenhouse gas balance using crop- and soil-based approaches: Two case studies. *Sci. Total Environ*. **2013**, 456, 299–306.

Pishgar-Komleh, S.H.; Omid, M.; Heidari, M.D. On the study of energy use and GHG (greenhouse gas) emissions in greenhouse cucumber production in Yazd province. *Energy* **2013**, 59, 63–71

Williams, A.G.; Audsley, E.; Sandars, D.L. Determining the environmental burdens and resource use in the production of agricultural commodities. Final report to Defra on project ISO205. **2006**. Available on www.agrilca.org and www.defra.gov.uk.

Yue, S. Optimum Nitrogen Management for High-yielding Wheat and Maize Cropping System. Ph.D. Thesis, China Agricultural University, Beijing, China, **2013**.

Zhang, W.; Dou, Z.,; He, P.;Ju, X.; Powlson, D.; Chadwick, D.; Norse, D.; Lu, Y.; Zhang, Y.; Wu, L.; Chen, X.; Cassman, K.G.; Zhang, F. New technologies reduce greenhouse gas emissions from nitrogenous fertilizer in China. *P. Natl. Acad. Sci. USA.* **2013**, 110, 8375–8380.

Zhang, W. Greenhouse gas emissions and reactive nitrogen losses assessment, mitigation potentials and management approaches of maize production in China. Ph.D. Thesis, China Agricultural University, Beijing, China, **2019**.
